# Involvement of the Circadian Rhythm and Inflammatory Cytokines in the Pathogenesis of Rheumatoid Arthritis

**DOI:** 10.1155/2014/282495

**Published:** 2014-05-08

**Authors:** Kohsuke Yoshida, Teppei Hashimoto, Yoshitada Sakai, Akira Hashiramoto

**Affiliations:** ^1^Faculty of Health Sciences, Kobe University School of Medicine, Kobe 654-0142, Japan; ^2^Department of General Internal Medicine, Kobe University School of Medicine, Kobe 650-0017, Japan; ^3^Division of Rehabilitation Medicine, Kobe University Graduate School of Medicine, Kobe 650-0017, Japan; ^4^Clinical Immunology, Kobe University Graduate School of Health Sciences, 7-10-2, Tomogaoka, Suma, Kobe 654-0142, Japan

## Abstract

Among the symptoms of patients with rheumatoid arthritis (RA), joint stiffness is influenced by diurnal rhythm and reaches peak in the morning, which is a common complaint and reflects the circadian nature of disease manifestation. In addition, inflammatory cytokines, which reach peak secretion early in the morning are major players causing the morning stiffness. In this review, we explore the link between the circadian clock and inflammation, focusing on the interactions of various clock genes with the immune-pathways underlying the pathology of rheumatoid arthritis.

## 1. The Biological Origins and Regulation of the Body Clock

The word “circadian” is defined as the period of physiological function and behavior of the organism, which is about 24 hours in an environment with no external constraints. The circadian rhythm has been shown to be involved in a number of physiological processes, including sleep/awakening, body temperature regulation, hormone secretion, division and proliferation of cells, and gastrointestinal function. Although we normally think of the circadian rhythm as 24 hours, it is in fact slightly longer in humankind [1, 2]. As a result, organisms need to correct their biological clock daily using external cues, the most effective of which is light stimulus. While other organisms use the same organ for both light reception and setting of the biological clock [3, 4], mammals have evolved separate locations for these two functions. The controlling center for rhythm oscillation in mammals is the hypothalamic suprachiasmatic nucleus (SCN), located at the base of the brain in front of the pituitary gland. The SCN is too deep in the brain to sense light cues directly and instead receives external cues via the optic nerve that transmits signals from the retina and combines the function of both light-dark tuning and rhythm transmission to act as a master clock of the whole body [5].

The role of the SCN in coordinating body rhythm was first shown in rodents, where removal of the SCN was observed to cause a collapse in the rhythmicity of behavior and endocrine activity, suggesting that this structure may be the underlying circadian pacemaker for physiological and behavioral activities in mammals [6]. However, subsequent studies found that the liver and lung cells maintain their own rhythm when cultured outside the body and without input from the SCN. Thus, it has become apparent that tissues and cells can provide their own peripheral rhythm, similar to that provided by the brain, which is maintained by the activities of the clock genes [6, 7].

The clock genes manage rhythm and time in a dual and hierarchical manner. The rhythm signals propagated from SCN are subject to a feedback loop provided by core clock genes including* CLOCK* (circadian locomotor output cycles kaput),* BMAL*1 (brain and muscle ARNT-like-1),* PER* (period) and* CRY* (cryptochrome), and orphan nuclear hormone receptors* REV-ERB* alpha (encoded by* Nr1d1*) and* Ror alpha*. The circadian expression of these genes is regulated through E/E′ boxes (CACGTG/CACGTT sequence), REV-ERB**α**/ROR response element (RRE), and DBP/E4BP4 binding element (D box) in their promoter regions. First, the CLOCK and BMAL1 proteins form a complex that binds to the E/E′ boxes present in the promoter regions of the* Per*1–3,* Cry*1-2,* Nr1d*1, and* Ror alpha* genes, thus regulating their transcription. The PER and CRY proteins, in turn, form a complex in the cytoplasm and relocate to the nucleus where they inhibit the genes induced by the CLOCK/BMAL1 protein complex, including their own transcription [6]. Second, REV-ERB and ROR compete on RRE element on the promoter region of* BMAL*1 to regulate its transcription [8]. In addition, DBP (D site of albumin promoter binding protein) promotes and E4BP4 (E4-binding protein 4) suppresses the transcription of* PER* by binding to the D box present in the promoter region [9, 10]. Although not all clock genes directly respond to CLOCK/BMAL1, this core control mechanism is repeated in about a 24-hour period. Importantly, circadian transcriptional circuits are governed by two design principles [11]: regulation of E/E′ boxes and RRE element follows a repressor-precedes-activator pattern, resulting in delayed transcriptional activity, whereas regulation of D box follows a repression-antiphasic-to-activator mechanism, which generated high-amplitude transcriptional activity. In addition, recent study has shown that circadian modulation of RNA polymerase II recruitment and chromatin remodeling occurs on a genome-wide scale [12], so that there are various mechanisms for generating rhythmicity of gene expression.

## 2. Melatonin and Inflammatory Cytokines Tune the Circadian Regulation

In the daytime, light-initiated signals are transmitted via the SCN to the pineal gland through the upper cervical ganglion. Melatonin, a hormone that mediates circadian rhythm adjustment, is produced by the pineal gland at night. Light stimulus causes an increase in the secretion of cortisol, serotonin, and dopamine, while suppressing melatonin, norepinephrine, and acetylcholine. Serum melatonin levels are normally undetectable in the daytime but are significantly higher during the night, in the absence of optical stimulation [13–16].

How might melatonin be involved in the pathogenesis of rheumatoid arthritis? As compared with healthy subjects, melatonin secretion at midnight is significantly increased in RA patients [17], and melatonin serum levels in the morning are higher in RA patients with shorter disease duration [18]. Inflammatory cytokines including IFN-**γ** (interferon-gamma), IL (interleukin)-1, and IL-6 are all secreted from human peripheral blood mononuclear cells in response to melatonin stimulation, and in fact, melatonin is detected in RA synovium tissue macrophages and joint fluid [19]. These studies seem to suggest that melatonin has an adverse effect on arthritis; on the other hand, melatonin inhibits the activity of MMP (matrix metalloproteinase)-9, which is involved in joint destruction in RA patients [20]. Thus, further study is needed to determine the effects of melatonin on joint destruction.

Studies in recent years have helped elucidate the influence of inflammatory cytokines on circadian mechanisms. For example, activation of the immune system counteracts infection and increases resistance to pathogens by inducing slow wave sleep, presumably via the production of inflammatory cytokines such as TNF-**α**, IL-2, or IFN-**γ** that are known to induce such sleep [21]. Indeed, LPS (lipopolysaccharide), which is the major component of the outer membrane of Gram-negative bacteria, stimulus also uniformly increases the secretion of these cytokines. Conversely, immune cells exhibit enhanced proinflammatory responses and LPS-induced IL-6 release if the circadian rhythm is disrupted by an external change in the light-dark cycle [22]. Interestingly, LPS-dependent secretion of TNF-**α** is significantly higher at night compared to day and is further enhanced by melatonin stimulation. Through their ability to promote sleep, cytokines are involved in the generation of an inner rhythm that controls the secretion of growth hormone, prolactin, and cortisol [23].

How is the relation between sleep and rheumatoid arthritis that is well known to be a chronic inflammatory disease? RA patients often exhibit sleep disorders classified as a nocturnal awakening type, and this type of disorder is characterized by a significant reduction in sleep efficiency and a significant increase in waking periods after sleep onset. Questionnaire studies of patients with sleep disorders report a “decline in the quality of sleep in patients with RA” as quantified by the Pittsburgh Sleep Quality Index [24, 25], and “excessive somnolence trend during the day in patients with RA” by the Epworth sleepiness scale [26]. Further, increased RA disease activity has been reported to correlate with sleep disorders, and the correlation is somewhat stronger in women and is mitigated by age [27, 28]. In addition, shift work was associated with risk of RA in women [29]. This could be supported by the concept that the body clock not only impacts on arthritic symptoms but is also involved in the pathogenesis of RA [16].

In RA, a major source of proinflammatory cytokines is immune cells such as T cells and macrophages. Spleen, peripheral lymph nodes, and peritoneal fluid-derived macrophages operate autonomous circadian clockworks even ex vivo, and spleen cells secrete TNF-**α** and IL-6 in a circadian manner under the stimulation with bacterial endotoxin [16, 30]. Further, endotoxin-induced cytokine and chemokine production was significantly affected by BMAL1 and REV-ERB*α* expression. Since the nuclear receptors ROR**α** and REV-ERB**α** are key molecules that modulate* BMAL*1 transcription in the process of feedback-regulation of circadian genes,* REV-ERB*α** knockout condition represented the loss of circadian gating of endotoxin response, especially in the release of IL-6, through macrophages [31], and agonists or overexpression of REV-ERB**α** inhibited the expression of Ccl2, also named monocyte chemotactic protein-1 (MCP-1), by binding to their promoter regions in murine macrophage cell line [32]. In addition, REV-ERBs regulate their target genes by inhibiting the functions of distal enhancers that are selected by macrophage-lineage-determining factors, thereby establishing a macrophage-specific program of repression [33]. This relation between BMAL1 and inflammation was also demonstrated from different angles that* BMAL*1-deleted myeloid cells disrupted differentiation and circadian oscillation of inflammatory monocyte [34].

CD4 positive T cells possess a circadian oscillator that drives rhythmic responses to the stimuli, as manifested by altered cell proliferation and cytokine secretion [16]. Interestingly, it has been shown that T cell numbers and its reactivity were stable during daytime, whereas a significant increase was observed in the late evening and early morning hours ex vivo [35]. These results may explain the portion of etiology for “morning stiffness of joints,” a common complaint and one of the best indicators of the condition of RA patients, correlates with the secretion of TNF-**α**, IL-6, and IFN-**γ**, whose levels peak from midnight to early in the morning [36], and chronobiology-based approach has been tested and shown to improve the morning symptoms of RA [37, 38].

## 3. A Pathological Link between RA and the Clock Genes: Synovitis and Wee-1 Kinase

RA is a chronic polyarthritis condition that goes through repeated relapse and remission as the disease progresses. These cycles of elevated inflammation cause deformation and destruction of the joint and irreversible dysfunctions. All joints are affected by RA, large, medium and small. Once activated by inflammation, the mesenchymal cells lining the joint space (synovial cells) begin to proliferate, and inflammatory granulation tissue, called pannus, invades the bone and cartilage, leading to joint destruction. Growth factors, angiogenic factors, and inflammatory lymphocytes work together to promote pannus formation, and joint destruction follows the secretion of MMPs and migration of synovial cells [39, 40]. When we examine cytokine activities, we find that IL-1**β** and MMPs are deeply involved in osteoporosis of the joint and in the narrowing of the surrounding joint space seen in the early stages of disease onset. In the later stages, TNF-**α**, IL-1**β**, and IL-6 are produced and accumulate in the joint cavity as synovitis worsens. Finally, at the peak of arthritis, modification of the inflammatory response by IL-17 is observed [41].

However, lymphocytes are not present in the local pannus during the early stages of RA. In these early stages, joint destruction is promoted by synovial cells under the influence of various cell cycle regulators and transcription factors. For example, synovial cell proliferation is enhanced through the suppression of p21 and overexpression of the transcription factor c-fos [42], while mitotic activity is inhibited through wee-1 kinase [43]. This is a characteristic feature of synovial cells, representing “tumor cell-like proliferation.” Indeed when* c-fos* transgenic mice are used in an arthritis model, joint destruction proceeds not as a result of lymphocyte invasion, but by synovial cell proliferation [44]. And, as reported earlier, the expression wee-1 kinase, a G2 /M cell cycle control factor, is increased in mice lacking an essential mammalian timekeeping gene* CRY* [45].

## 4. Clock Genes and Arthritis; Cry, Per and TNF-***α*** Exacerbate Inflammation

To verify the significance of increased wee-1 expression in* CRY* knockout mice (*CRY*1^−/−^ *CRY*2^−/−^ mice), we utilized a mouse model of arthritis [46]. First, we confirmed the influence of arthritis on clock gene expression using wild type (WT) mice that had been administered anticollagen antibody and LPS and PER2 protein levels in the synovium were monitored. PER2 is usually expressed at night, but in the arthritis model PER2 was highly expressed in the morning. In addition, the phase of* PER*1/2 mRNA expression in spleen lymphocytes was shifted back ~6 hrs, and* BMAL*1 and* Per*1/2 mRNA expression levels were reduced. These observations indicate that the onset of arthritis indeed affects the expression of clock genes* in vivo*.

Next, we examined spleen-derived lymphocytes from* CRY* knockout mice. Peripheral T lymphocytes were found to be constitutively activated, and stimulation of splenocytes by anti-CD3/CD28 antibodies produced higher amounts of TNF-**α**. In addition, wee-1 protein was overexpressed in the spleens of* CRY* knockout mice. Together, these results suggest that* CRY* knockout mice are ready or primed for arthritis onset. Accordingly, arthritis of the limbs was strongly induced by type *ΙΙ* collagen cocktail in* CRY* knockout mice, and this arthritis was suppressed by anti-TNF-**α** antibodies. Finally, mutual regulation between the TNF-**α** and CRY genes was demonstrated using luciferase reporter assays.

In addition, recent studies have shown that the absence of* CRY* leads to constitutive activation of protein kinase A, which results in phosphorylation of p65, and thereby ultimately induces NF-*κ*B activation and expression of IL-6 as well as TNF-**α** [47]. Consistently, expression of* CRY*1 was markedly decreased by administration of melatonin, subsequently aggravated in mouse antitype *ΙΙ* collagen antibody-induced arthritis [48]. Further, a phase and amplitude of the clock genes were different between osteoarthritis fibroblast-like synovial cells (OA-FLS) and RA-FLS cells [49]. Thus, these results suggest that clock genes, such as CRY, seem to be deeply involved in both inflammation and arthritis.

Then, can the inflammatory pathways directly affect the expression of clock genes? Table 1 shows the effect of TNF-**α** on expression of clock genes in mouse and human cells. Using NIH3T3 mouse fibroblast cells, Cavadini et al. reported TNF-**α** inhibited the expression of* PER*1/2/3,* DBP*,* TEF,* and* HLF*, slightly enhanced* CLOCK* and* REV-ERB*α**, and did not affect* BMAL*1, by interfering with E box-mediated transcription of clock genes. And also IL-1**β**, but not IL-6, seemed to have a biological effect as well as TNF-**α** [50]. Further, the early effect of TNF-**α** on expression of the* PER*1 gene is dependent on p38, mitogen-activated protein kinase (MAPK), and/or calcium signaling, whereas its effect on the* DBP* gene is independent of MAPK but dependent on calcium signaling [51]. In human monocytic THP-1 cells, TNF-**α** enhanced the expression of* BMAL*1 and* CRY*2, whereas it decreased those of* PER*2,* CRY*1, and* REV-ERB*α** [52]. Unlike mouse cells, TNF-**α** did not affect or slightly enhanced the expression of* PER*1 in OA-FLS, RA-FLS, and THP-1 cells [49, 52, 53]. However, TNF-**α** has an inhibitory effect on the expression of DBP and* PER*3 in OA-FLS cells [49]. And we observed, using RA-FLS cells, TNF-**α** also inhibited the expression of* PER*2 and slightly enhanced CLOCK as well, whereas it significantly increased the expression of* BMAL*1 and* Cry*1 [53]. Interestingly, in TNF-**α**-stimulated RA-FLS cells, expression of* DBP*,* TEF* and* HLF* was reduced while* E4BP4* was increased, a positive and negative transcriptional regulator of* PER*2, respectively, suggesting another transcriptional regulation of* PER*2 by TNF-**α** via D box, but not E box [53]. Thus, the proinflammatory cytokines such as TNF-**α** could affect the expression of clock genes through their inflammatory cascades. In addition, if we recall that* PER*2 knockout mice exhibit increased resistance to apoptosis in thymocytes [54], decreased expression of* PER*2 by TNF-**α** can also contribute to the resistance of synovial cells to apoptosis and may contribute to the tumor-like growth of the synovium.

Recently, series of observations has made increasingly clear the involvement of clock genes in the pathogenesis of arthritis. Kouri et al. confirmed that BMAL1 protein is markedly localized in the cytoplasm of RA synovium [49]. Interestingly, in mouse model of osteoarthritis (OA), circadian oscillations of* PER*2 transcription are reported to be significantly reduced in cartilage from aged mice [55], and the robust oscillations of* PER*2 transcription are confirmed in cartilage from juvenile mice [56]. These results suggest not only the similarity to human OA that affected individuals increases with age, but also the relation between circadian rhythms and physiology of cartilage formation, that is, also important for joint destruction mechanism in RA.

## 5. Conclusion

The past 20 years have seen dramatic progress in elucidating the genetic regulation of the body's circadian rhythm. An intricate system of intermolecular interaction modulates the circadian rhythms in cells to an approximate 24-hour cycle, and this cycle is normally maintained and readjusted by both central and peripheral mechanisms. Maintenance of this cycle is important in maintaining a number of homeostatic functions in the body, and dysfunction of this body clock can promote a number of human diseases and pathologies. Since clock genes are highly conserved, it is not surprising that they are responsible for modulating a large number of biological functions. We expect that future research will help us understand not only the various functions of clock genes, but also the strategy to save the primary roles and functions of circadian clock for the human health which is disturbed by a number of factors in the modern society.

## Figures and Tables

**Table 1:** Effect of TNF-*α* on the expression of clock genes in mouse NIH3T3, human OA-FLS, RA-FLS, and THP-1 cell line*.

Clock gene	Mouse	Human		
		Fibroblast		Monocyte
	NIH3T3	OA-FLS	RA-FLS	THP-1
*Bmal1 *	Not affected	—	Up	Up
*Clock *	Slightly up	—	Slightly up	Not affected
*Per1 *	Down (early~3 h: up)	Not affected	Slightly up	Slightly up
*Per2 *	Down	—	Down	Down
*Per3 *	Down	Down	—	—
*Cry1 *	Up	—	Up	Down
*Cry2 *	Slightly down	—	Not affected	Up
*Dbp *	Down	Down	Down	—
*Tef *	Down	—	Down	—
*Hlf *	Down	—	Down	—
*E4BP4 *	—	—	Up	—
*Re* *v*-*erbα*	Slightly up	—	—	Down

*Based on results of the refer 49–53. — denotes no data. OA-FLS: osteoarthritis fibroblast-like synovial cells. RA-FLS: rheumatoid arthritis fibroblast-like synovial cells.
